# Regulatory Effects of Exogenous Trehalose on the Growth and Photosynthetic Characteristics of Celery (*Apium graveolens* L.) Under Salt Stress

**DOI:** 10.3390/plants15020212

**Published:** 2026-01-09

**Authors:** Yanqiang Gao, Liangmei Zhang, Wenjing Rui, Miao Zhang, Zixiao Liang, Kaiguo Pu, Youlin Chang, Yongwei Ma, Jingwen Huo, Jiongjie Zhang, Jing Li, Jianming Xie

**Affiliations:** College of Horticulture, Gansu Agricultural University, Lanzhou 730070, China; zlm20000712@163.com (Y.G.); 17793295093@163.com (L.Z.); ruiwenjin@163.com (W.R.); zm15609379816@163.com (M.Z.); liangzixiao1998@163.com (Z.L.); pukaiguo1998@163.com (K.P.); changyoulin98@163.com (Y.C.); 1073324020441@st.gsau.edu.cn (Y.M.); 13659171492@163.com (J.H.); 15101343103@163.com (J.Z.)

**Keywords:** salt stress, trehalose, celery, growth, photosynthetic characteristics

## Abstract

Salinity has been recognized as one of the major environmental stresses that restrict the growth and quality of celery (*Apium graveolens* L.). Therefore, this study investigates the impact of different NaCl concentrations on celery growth and photosynthetic characteristics, as well as the potential regulatory role of exogenous trehalose application in mitigating the stress-induced effects. The results indicated that an increase in NaCl concentration from 50 to 200 mM markedly inhibited the growth of celery plants compared to that under control conditions. The application of different concentrations of trehalose mitigated the inhibitory effects of salt stress (100 mM NaCl) on celery growth and photosynthesis. Among the different trehalose treatments, T3 (10 mM trehalose) exhibited the most significant effects, increasing the aboveground biomass, belowground biomass, plant height, chlorophyll a, chlorophyll b, total chlorophyll, and net photosynthetic rate compared to that of salt stress alone, respectively. Furthermore, trehalose treatments enhanced the various fluorescence parameters, including the maximum efficiency of PSII photochemistry (Fv/Fm), coefficient of photochemical quenching (qP), fluorescence intensity, and photosynthetic performance index (PIabs) under salt stress. Meanwhile, trehalose reduced intercellular carbon dioxide concentration, excess excitation energy (1-qP)/NPQ, heat dissipation per unit area (DIo/CSm), and energy dissipated per reaction center (DIo/RC). Additionally, the results of principal component analysis (PCA) and membership function comprehensive evaluation indicate that an appropriate concentration of trehalose positively alleviates the salnitiy-induced effects in celery. Overall, the T3 demonstrated the most promising effects on mitigating the effects of salt stress by decreasing the excess excitation energy of PSII in celery leaves through the heat dissipation pathway. This reduction lowers the excitation pressure on the reaction centers, enhances the activity of PSII reaction centers per unit cross-section, and improves photosynthesis activity, thereby improving the growth of celery plants under salt stress.

## 1. Introduction

Salt stress is one of the major environmental stresses that severely limits crop growth and yield [1]. Plants exposed to salt stress exhibit symptoms including osmotic stress, growth inhibition, [2] and weaken photosynthesis [3]. Salt stress has been widely recognized to negatively affect the growth, development, and reproductive biology of vegetables [4]. El-Beltagi et al. [5] observed that an increase in salinity (NaCl) level resulted in decreased the leaf area, stemand root length, and dry weight of both stems and roots in broccoli plants, adversely affecting the linear electron flow (LEF) and photosystem II activity. Khan et al. [6] demonstrated that salt stress negatively affected the photosynthetic efficiency of tomato, thereby influencing the overall plant growth and root morphology. Another study has shown a significant reduction in net photosynthetic rate (Pn), stomatal conductance (Gs), transpiration rate (Tr), the maximum quantum efficiency of PSII in dark-adapted state (Fv/Fm and Fv/Fo) in rice seedlings subjected to salt stress, sunsequently reducing the biomass yield [7]. Other studies have also reported similar observation in kidney beans and cucumber [8,9].

Trehalose (Tre) is a non-reducing disaccharide widely found in living organisms, and was discovered in the 19th century by Wiggers from ergot in rye [10]. Trehalose metabolism is of great significance for plants to respond to abiotic stress. It is an important signaling molecule and cell protective agent. Meanwhile, an appropriate concentration of trehalose can reduce Na ion accumulation and maintain ROS balance [10]. In recent years, it has received considerable attention as an effective osmotic agent capable of inducing salt tolerance in plants [11]. Previous studies indicate that trehalose can mitigate the inhibitory effects of salt stress on the growth of various plants, including Arabidopsis [12], rice [13], okra plants [14], and sunflower [15], and Yang et al. observed that 10 mM trehalose significantly slowed down the decline in tomato biomass caused by salinity stress, which was supported by enhanced chlorophyll fluorescence and rapid light response curve [16]. Exogenous application of trehalose was reported to enhance photosynthetic electron transport in corn leaves during heat stress, leading to significant increase inactual photosystem II efficiency [Y(II)] and photochemical quenching coefficient (qP) [17]. Additionally, trehalose application significantly improved drought tolerance in corn plants by upregulating photosynthetic and water-related properties [18]. Trehalose enhances the photochemical efficiency and electron transport rate of PSII in winter wheat subjected to high temperature and drought stress [19]. Garcia et al. [20] reported that rice seedlings exposed to salt stress in trehalose medium maintained high root cell integrity and cell division. However, the potential positive effects of trehalose may vary based on the concentration used, plant species, and growing conditions, necessitating the identification of appropriate concentrations under various types of abiotic stress.

Celery (*Apium graveliens* L.) is a cool-season vegetable, which is rich in vitamins, minerals, essential amino acids, and dietary fiber [21]. It also exhibits pharmacological effects, including lowering blood pressure, regulating blood lipids, and antioxidant properties [22]. With the improvement in living standards, there is an increasing demand for celery, particularly of high-quality. Celery roots exhibit a short and shallow morphology, which significantly limits efficiency of water absorption and results in low tolerance for salt stress, thereby presenting a major challenge to the growth and quality of celery [23]. Song et al. [22] reported that celery plants treated with NaCl exhibited salt toxicity, characterized by stunted growth and reduced photosynthetic capacity. Despite significant advancements in understanding the regulatory role of trehalose in crop responses to abiotic stresses, there is limited research on examining the effects of exogenous trehalose on celery growth and photosynthesis. What are the effects of salt stress on the growth and photosynthesis of celery? Can trehalose positively alleviate the salt stress of celery? Therefore, it is important to study the effect of salt stress on the growth and photosynthesis of celery and whether trehalose alleviates salt stress. In this context, a preliminary experiment was conducted to evaluate the effects of different salt concentrations (NaCl; 0, 50, 100, 150, and 200 mM) on the growth of celery plants. Subsequently, different concentrations of exogenous trehalose (0, 1, 5, 10, 15 and 20 mM) were assessed to determine their potential positive effects on regulating the growth and photosynthetic characteristics of celery plants under moderate stress (100 mM NaCl). This study aims to establish a basis for the potential application of trehalose in alleviating salinity-induced effects in vegetables and to facilitate the expansion of celery cultivation in salt affected areas to increase productivity.

## 2. Results

### 2.1. Effects of Different NaCl Concentrations on Celery Growth and Root Morphology

**Figure 1:**
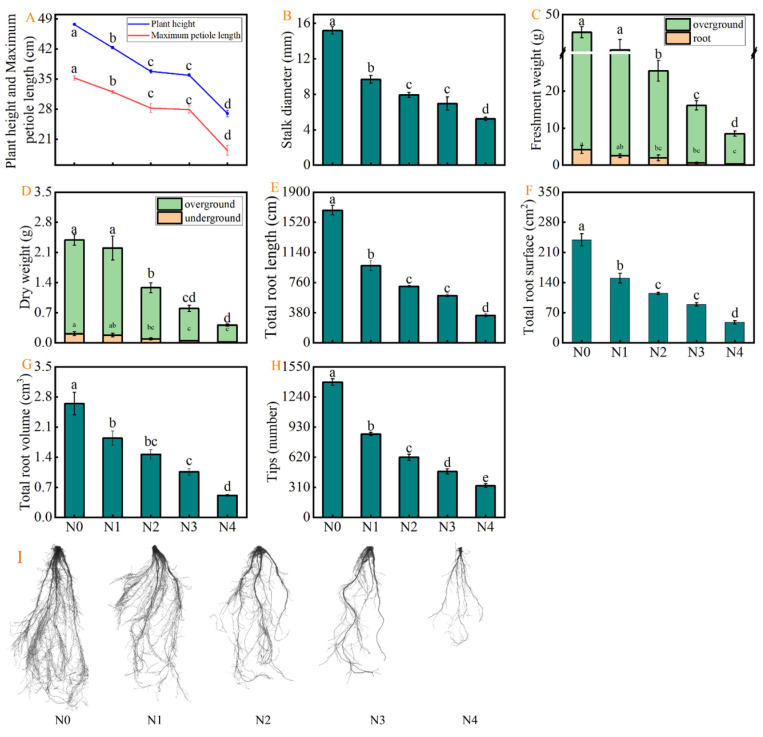
Effects of salt stress on (**A**) plant height and maximum petiole length, (**B**) stalk diameter, (**C**) fresh weight, (**D**) dry weight, (**E**) total root length, (**F**) total root surface area, (**G**) total root volume, (**H**) tips, and (**I**) root morphological characteristics of celery plants. The results are expressed as the mean + SE of three replicates, and the different letters denote the significant difference among treatments (*p* < 0.05), according to Duncan’s multiple tests. N0, 0 mM NaCl. N1, 50 mM NaCl. N2, 100 mM NaCl. N3, 150 mM NaCl. N4, 200 mM NaCl.

Figure 1 demonstrates the effects of different NaCl concentrations on the growth and biomass of celery plants. An increase in NaCl concentrations significantly decreased the plant height, petiole length, stalk diameter, and biomass compared to that of control (N0). The plant height of celery under N1, N2, N3, and N4 treatments was significantly reduced by 11.32%, 22.85%, 24.74%, and 43.40%, compared to that of N0, respectively. Likewise, the maximum petiole length was decreased by 9.26%, 19.85%, 20.79%, and 47.83%, whereas the stalk diameter was reduced by 36.25%, 47.81%, 54.14%, and 65.60%, compared to N0, respectively (Figure 1A,B). Salt stress significantly reduced the biomass of celery plants, with N2, N3, and N4 treatments leading to a decrease in fresh weight of aboveground parts by 42.68%, 62.03%, and 80.08%, and in the fresh weight of belowground parts by 53.17%, 85.71%, and 91.27%, compared to N0, respectively. Likewise, the N2, N3, and N4 treatments decreased the dry weight of aboveground parts by 45.46%, 65.54%, and 82.05%,while that of root parts by 56.92%, 78.38%, and 90.78%, compared to N0, respectively (Figure 1C,D). In addition, the root morphological images further confirmed the inhibitory effect of different NaCl concentrations on celery root systems. Compared to N0, the total root length, total root volume, number of root tips, and total root surface area were markedly decreased by N1, N2, N3, and N4 treatments (Figure 1E–I).

### 2.2. The Effect of Different Trehalose Concentrations on the Growth and Root Morphology of Celery Under Salt Stress

**Figure 2:**
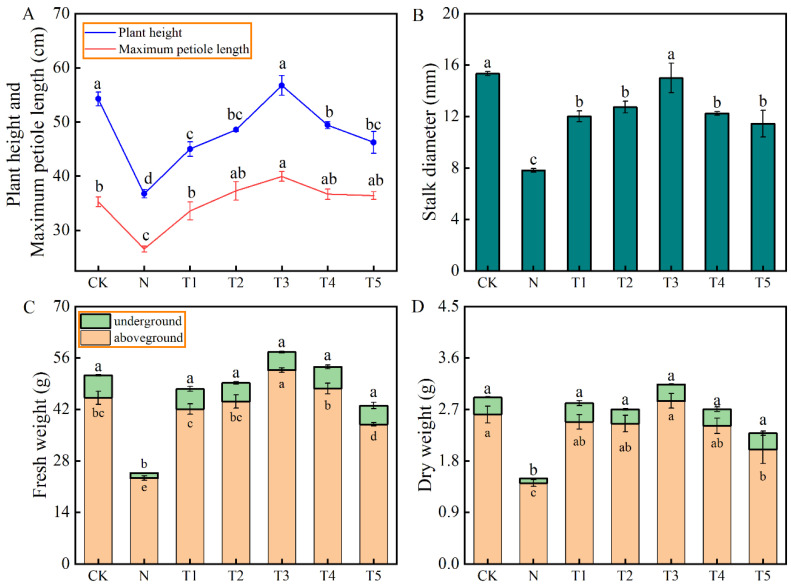
Effects of different concentrations of trehalose on (**A**) plant height and maximum petiole length, (**B**) stem diameter, (**C**) fresh weight, and (**D**) dry weight of celery under salt stress. The application of trehalose and salt is not simultaneous; salt stress is carried out after the application of trehalose. The results are expressed as the mean + SE of three replicates, and the different letters denote thesignificant difference among treatments (*p* < 0.05), according to Duncan’s multiple tests. CK, 0 mM Tr + 0 mM NaCl. N, 0 mM Tr + 100 mM NaCl. T1, 1 mM Tr + 100 mM NaCl. T2, 5 mM Tr + 100 mM NaCl. T3, 10 mM Tr + 100 mM NaCl. T4, 15 mM Tr + 100 mM NaCl and T5, 20 mM Tr + 100 mM NaCl.

Figure 2 and Table 1 illustrate the impact exogenous trehalose application on the growth and root morphological parameters of celery under salt stress. Salt stress (N treatment) significantly decreased the plant height, maximum petiole length, stalk diameter, dry and fresh weight, and root morphology parameters of celery plants compared to CK. However, the application of different concentrations of trehalose significantly improved the growth attributes of celery plants under salt stress, but at varying degrees. An initial increase followed by a subsequent decreasing trend in growth improvement was observed with the increase in the trehalose concentrations (Figure 2A–D, Table 2). The T1, T2, T3, T4, and T5 treatments significantly increased the plant height by 22.5%, 32.21%, 54.45%, 34.48%, and 25.86%, compared to N, respectively. Similarly, these treatments resulted in increases in maximum petiole length by 26.51%, 40.45%, 50.50%, 38.19%, and 37.19%, and stalk diameter by 54.01%, 63.15%, 91.97%, 56.79%, and 46.67%, compared to that of N treatment, respectively (Figure 2A,B). The fresh and dry weight of celery exhibited a similar trend (Figure 2C,D). Compared to CK, salt stress reduced the total root length, total root surface area, total root volume, and tips and branching by 59.11%, 55.20%, 51.17%, 63.74%, and 74.65%, respectively. On the other hand, the salt stress-induced effects on total root length, total surface area, and tips and branching were significantly alleviated by T1, T2, T3, and T4 treatments (Table 1).

### 2.3. Correlation, Principal Component, and Membership Function Analysis for Growth and Root System Morphological Indicators of Celery

**Figure 3:**
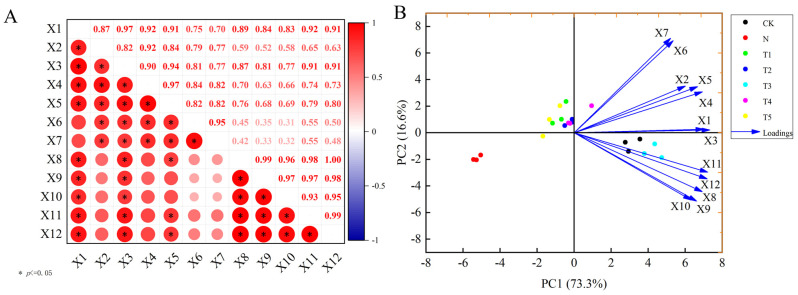
(**A**) Correlation analysis and (**B**) Principal component analysis for morphological indicators of celery. “X1”, “X2”, “X3”, “X4”, “X5”, “X6”, “X7”, “X8”, “X9”, “X10”, “X11”, “X12” represent plant height, maximum leaf petiole length, stalk diameter, aboveground fresh weight, aboveground dry weight, belowground fresh weight, belowground dry weight, total root length, total root surface area, total root volume, tips and branching, respectively. The application of trehalose and salt is not simultaneous; salt stress is carried out after the application of trehalose. CK, 0 mM Tr + 0 mM NaCl. N, 0 mM Tr + 100 mM NaCl. T1, 1 mM Tr + 100 mM NaCl. T2, 5 mM Tr + 100 mM NaCl. T3, 10 mM Tr + 100 mM NaCl. T4, 15 mM Tr + 100 mM NaCl and T5, 20 mM Tr + 100 mM NaCl.

The multivariate analysess were performed to comprehensively reveal the effect of trehalose application on celery growth under salt stress (Figure 3A,B, Table 2). The correlation analysis demonstrated significant relationships among various parameters of salt-stressed celery with exogenous trehalose application (Figure 3A). Principal component analysis clearly differentiated the salt stress (N treatment) from other treatments, indicating that different concentrations of trehalose positively regulated the growth of celery under salt stress. The PC1 and PC2 explained about 73.3% and 16.6% of the total variance, with the primary contributing growth indicators for PC1 were X1 and X2, while those for PC2 were X6 and X7 (Figure 3B). Furthermore, the comprehensive evaluation analysis for growth indices using the affiliation function method resulted in the following ranking: T2, T3, T4, T5, CK, N, and T1 (Table 2).

### 2.4. Effect of Different Concentrations of Trehalose on Photosynthetic Pigments and Gas Exchange Parameters of Celery Under Salt Stress

**Figure 4:**
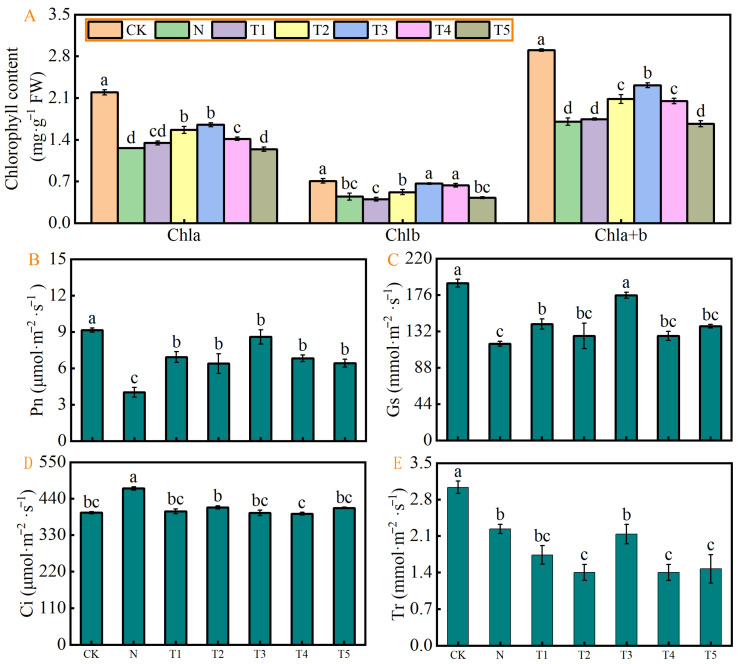
Effects of different concentrations of trehalose on (**A**) chlorophyll content; (**B**) net photosynthetic rate, Pn; (**C**) stomatal conductance, Gs; (**D**) Intercellular CO_2_ concentration, Ci; and (**E**) transpiration rate, Tr Of celery plant under salt stress. The application of trehalose and salt is not simultaneous; salt stress is carried out after the application of trehalose. The results are expressed as the mean + SE of three replicates, and the different letters denote thesignificant difference among treatments (*p* < 0.05), according to Duncan’s multiple tests. CK, 0 mM Tr + 0 mM NaCl. N, 0 mM Tr + 100 mM NaCl. T1, 1 mM Tr + 100 mM NaCl. T2, 5 mM Tr + 100 mM NaCl. T3, 10 mM Tr + 100 mM NaCl. T4, 15 mM Tr + 100 mM NaCl and T5, 20 mM Tr + 100 mM NaCl.

Figure 4 shows the effects of exogenous trehalose on the photosynthetic pigment content and photosynthetic parameters of celery under salt. Results showed that salt stress (N) significantly decreased the Chl a, Chl b, total Chl, Pn rate, Gs, and Tr by 42.64%, 37.24%, 41.33%, 56.00%, 38.53%, and 26.83%, while significantly increased Ci by 18.46% compared to CK, respectively (Figure 4A–E). The trehalose treatments (T1, T2, T3, T4, and T5) significantly increased the chlorophyll and photosynthetic parameters to varying degrees under salt stress (Figure 4A–E). The effects of T1–T4 were more pronounced on chl a, b, and Pn, that of T2 on Tr, and T1 and T3 on Gs.

### 2.5. Effect of Different Concentrations of Trehalose on Chlorophyll Fluorescence Parameters in Celery Leaves Under Salt Stress

**Figure 5:**
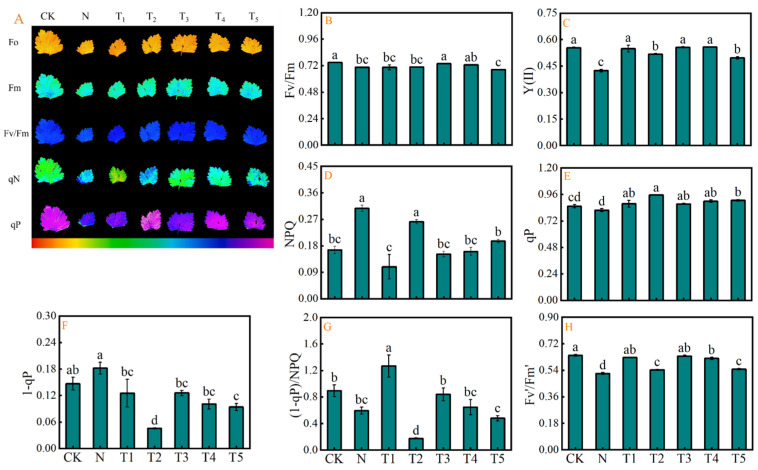
Effects of different concentrations of trehalose on (**A**) Fo, Fm, Fv/Fm, qN and qP, (**B**) maximum efficiency of PSII photochemistry, (Fv/Fm), (**C**) actual photochemical efficiency of PSII, [Y(II)], (**D**) non-photochemical quenching, (NPQ), (**E**) coefficient of photochemical quenching, (qP), (**F**) excitation pressure of PSII, (1-qP), (**G**) excess excitation energy, (1-qP)/NPQ, and (**H**) efficiency of excitation energy capture by open PSII reaction centers, (Fv′/Fm′) of celery leaves under salt stress. The application of trehalose and salt is not simultaneous; salt stress is carried out after the application of trehalose. The results are expressed as the mean + SE of three replicates, and the different letters denote the significant difference among treatments (*p* < 0.05), according to Duncan’s multiple tests. CK, 0 mM Tr + 0 mM NaCl. N, 0 mM Tr + 100 mM NaCl. T1, 1 mM Tr + 100 mM NaCl. T2, 5 mM Tr + 100 mM NaCl. T3, 10 mM Tr + 100 mM NaCl. T4, 15 mM Tr + 100 mM NaCl and T5, 20 mM Tr + 100 mM NaCl.

Figure 5 illustrates the impact of exogenous trehalose application and salinity stress on chlorophyll fluorescence parameters in celery leaves. In addition, the chlorophyll fluorescence imaging of Fo, Fm, Fv/Fm, qN, and qP is depicted in Figure 5A. Results indicated that salt stress significantly decreased the Fv/Fm, Y(II), qP, and Fv′/Fm′ indices by 5.62%, 23.39%, 4.10%, and 19.15%, respectively, while increased the NPQ, 1-qP, and (1-qP)/NPQ compared to CK (Figure 5B–H). The exogenous application of trehalose mitigated the stress induced effects, with T3 enhancing the Fv/Fm, while T1–T5 treatments improved the Y(II), qP, and Fv′/Fm′. In addition, the T3–T5 treatments resulted in a decrease in NPQ, while T1–T5 led to a reduction in 1-qP compared to salt stress N treatment. Additionally, T2 treatment decreased the (1-qP)/NPQ compared to N treatment.

### 2.6. Multivariate Analysis for the Relationships of Photosynthetic Parameters and Chlorophyll Fluorescence Parameters of Celery

**Figure 6:**
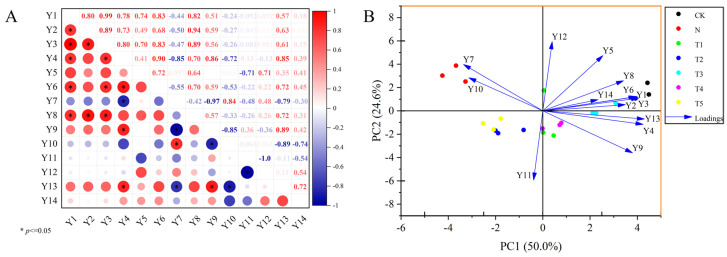
(**A**) Correlation analysis of photosynthetic parameters and chlorophyll fluorescence parameters, (**B**) Principal component analysis of photosynthetic parameters and chlorophyll fluorescence parameters. Y1, Y2, Y3, Y4, Y5, Y6, Y7, Y8, Y9, Y10, Y11, Y12, Y13, and Y14 represent Chl a, Chl b, total Chl, Pn, Tr, Gs, Ci, Fv/Fm, Y(II), NPQ, qP, 1-qP, Fv′/Fm′ and (1-qP)/NPQ, respectively. The application of trehalose and salt is not simultaneous; salt stress is carried out after the application of trehalose. CK, 0 mM Tr + 0 mM NaCl. N, 0 mM Tr + 100 mM NaCl. T1, 1 mM Tr + 100 mM NaCl. T2, 5 mM Tr + 100 mM NaCl. T3, 10 mM Tr + 100 mM NaCl. T4, 15 mM Tr + 100 mM NaCl and T5, 20 mM Tr + 100 mM NaCl.

The regulatory role of trehalose in the photosynthetic characteristics of celery under salt stress was elucidated through correlation analysis, PCA, and a comprehensive evaluation of membership functions. Correlation analysis indicated highly significant and positive correlations between Chl a and total Chl and Y3 in celery (r^2^ = 0.99), while there was a highly significant negative correlation between Ci and Y(II) (r^2^ = −0.97) (Figure 6A). PC1 and PC2 effectively differentiated the salt stress treatment from trehalose treatments, explaining 50.0% and 24.6% of the total variance, respectively. The primary growth indicators for PC1 were Chl b, total chl, whereas for PC2 was Fv′/Fm′ (Figure 6B). Furthermore, the comprehensive evaluation of indicators related to phototrophic characteristics was conducted using the affiliation function method, resulting in the following order: CK, T4, T1, N, T5, T3 and T2 (Table 3).

### 2.7. Effect of Different Concentrations of Trehalose on the Chlorophyll Fluorescence Dynamics (OJIP) Curve and JIP-Test Parameters of Celery Leaves Under Salt Stress

**Figure 7:**
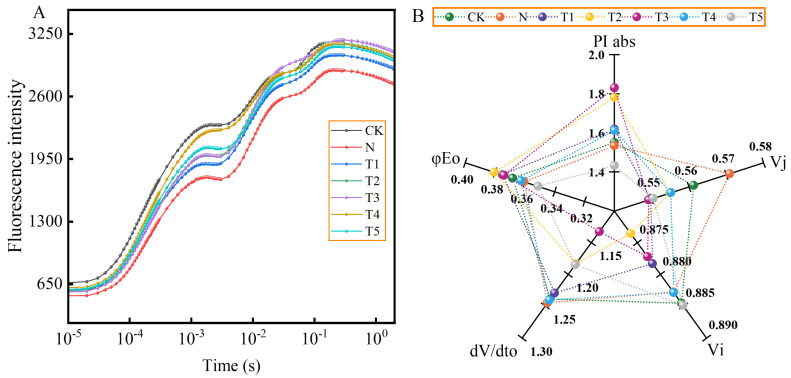
Effects of different concentrations of trehalose on (**A**) chlorophyll fluorescence dynamics (OJIP) curve, and (**B**) JIP-test parameters of celery under salt stress. The application of trehalose and salt is not simultaneous; salt stress is carried out after the application of trehalose. CK, 0 mM Tr + 0 mM NaCl. N, 0 mM Tr + 100 mM NaCl. T1, 1 mM Tr + 100 mM NaCl. T2, 5 mM Tr + 100 mM NaCl. T3, 10 mM Tr + 100 mM NaCl. T4, 15 mM Tr + 100 mM NaCl and T5, 20 mM Tr + 100 mM NaCl.

Figure 7 illustrates the effects of exogenous trehalose application on fluorescence intensity and JIP-test parameters in celery leaves under salt stress. Compared to CK, the fluorescence intensity was reduced under salt stress. Furthermore, salt stress increased the Vj and dV/dto while decreasing photosynthetic performance index (PIabs) compared to that of CK. However, the different trehalose treatments enhanced the fluorescence intensity to varying degrees compared to that of salt stress alone (Figure 7A). The T1–T5 treatments resulted a reduction in the Vj and dV/dto values, whereas T1–T3 significantly decreased the Vi values. Conversely, the φEo values for T1–T3 increased, and the absolute PI values for T1–T4 increased (Figure 7B).

### 2.8. Effect of Different Concentrations of Trehalose on Energy Distribution per Unit Area and Leaf Specific Activity Parameters of Celery Leaves Under Salt Stress

**Figure 8:**
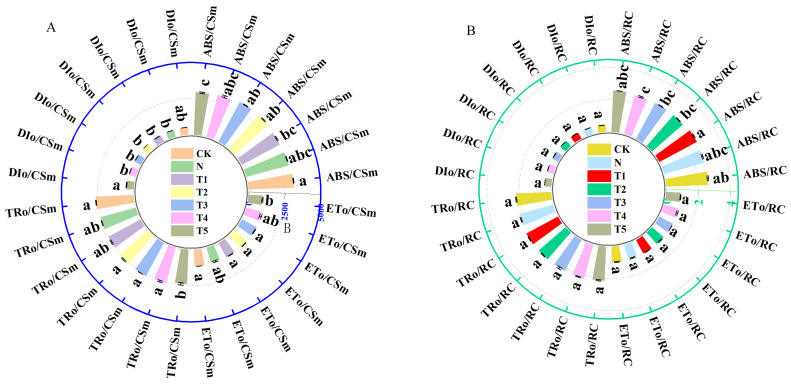
Effects of different concentrations of trehalose on (**A**) energy distribution per unit area of leaf blade and (**B**) blade specific activity parameter in celery under salt stress. The application of trehalose and salt is not simultaneous; salt stress is carried out after the application of trehalose. The results are expressed as the mean + SE of three replicates, and the different letters denote the significant difference among treatments (*p* < 0.05), according to Duncan’s multiple tests. CK, 0 mM Tr + 0 mM NaCl. N, 0 mM Tr + 100 mM NaCl. T1, 1 mM Tr + 100 mM NaCl. T2, 5 mM Tr + 100 mM NaCl. T3, 10 mM Tr + 100 mM NaCl. T4, 15 mM Tr + 100 mM NaCl and T5, 20 mM Tr + 100 mM NaCl.

Results showed significant effects of salt stress and trehalose treatments on the distribution and activity ratio per unit cross-sectional area of celery leaves (Figure 8). Salt stress decreased the ABS/CSm, TRo/CSm, ETo/CSm, ABS/RC, TRo/RC, and ETo/RC compared to CK (Figure 8A,B). The T2–T4 treatments increased the ABS/CSm and TRo/CSm, while T1–T4 enhanced the ETo/CSm and decreased DIo/CSm under salt stress (Figure 8A). Compared to salt stress, the ABS/RC and TRo/RC was greater with T1 treatment, TRo/RC with T1–T4, ETo/RC with T1–T3, while DIo/RC was lower with T2–T4 treatments (Figure 8B).

### 2.9. Predictions Analysis

**Figure 9:**
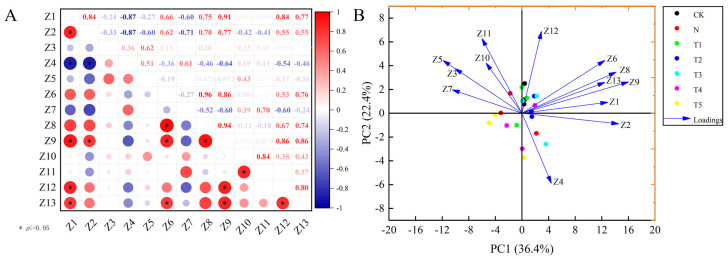
(**A**) Correlation analysis of fast chlorophyll fluorescence kinetic parameters, (**B**) principal component analysis of fast chlorophyll fluorescence kinetic parameters. Z1, Z2, Z3, Z4, Z5, Z6, Z7, Z8, Z9, Z10, Z11, Z12, and Z13 represent phi(Eo), PI (abs), Vj, Vi, dV/dto, ABS/CSm, DIo/CSm, TRo/CSm, ETo/CSm, ABS/RC, DIo/RC, TRo/RC, and ETo/RC, respectively. The application of trehalose and salt is not simultaneous; salt stress is carried out after the application of trehalose. CK, 0 mM Tr + 0 mM NaCl. N, 0 mM Tr + 100 mM NaCl. T1, 1 mM Tr + 100 mM NaCl. T2, 5 mM Tr + 100 mM NaCl. T3, 10 mM Tr + 100 mM NaCl. T4, 15 mM Tr + 100 mM NaCl and T5, 20 mM Tr + 100 mM NaCl.

Correlation analysis revealed positive relationships between Z1 and Z2, Z9, Z12, Z13; Z6 and Z8, Z9, Z13, and Z12 and Z13 in celery. Conversely, a negative correlation was observed between Z2 and Z4 (Figure 9A). PC1 and PC2 explain 36.4% and 22.4% of the total variance, respectively. The primary contributing growth indicators for PC1 were Z1 and Z9, whereas for PC2 was Z12 (Figure 9B). Furthermore, the comprehensive evaluation of linear electron transport characteristics in PSII was conducted using the affiliation function method, resulting in the following order: T5, T2, T3, T1, T4, CK and N (Table 4).

### 2.10. Ranking of the Combined Affiliation Function for All Indicators

**Figure 10:**
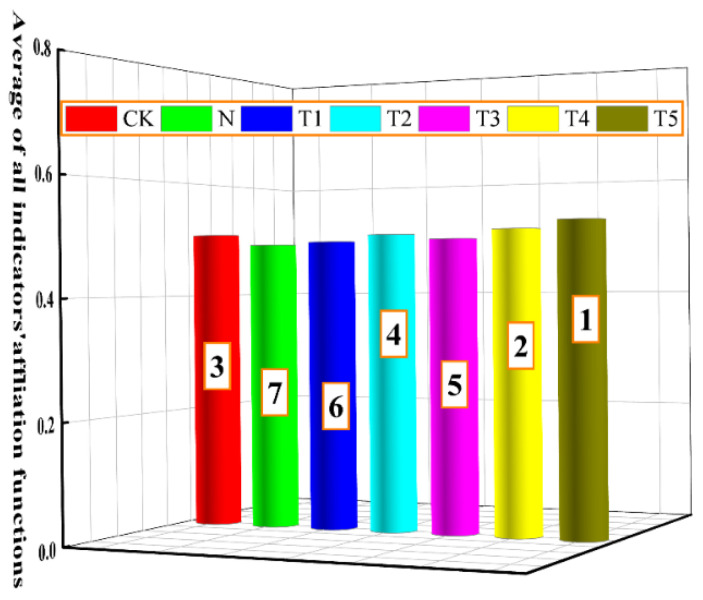
Ranking of the combined affiliation function for all indicators. The application of trehalose and salt is not simultaneous; salt stress is carried out after the application of trehalose. CK, 0 mM Tr + 0 mM NaCl. N, 0 mM Tr + 100 mM NaCl. T1, 1 mM Tr + 100 mM NaCl. T2, 5 mM Tr + 100 mM NaCl. T3, 10 mM Tr + 100 mM NaCl. T4, 15 mM Tr + 100 mM NaCl and T5, 20 mM Tr + 100 mM NaCl.

Figure 10 shows the combined attribution function for the evaluation results across all indicators. The treatment groups are ranked as: T5, T4, CK, T2, T3, T1, and N, indicating that different concentrations of trehalose alleviate salt stress induced effects in celery compared to that of salt stress alone. Figure 10. Ranking of the combined affiliation function for all indicators.

## 3. Discussion

### 3.1. The Inhibitory Effect of Salt Stress on Celery Growth

Salt stress represents a serious environmental constraint that severely impairs plant growth and development [24], negatively impacting root morphology and cell vitality [6]. Plant roots are the first organs to be affected by salt stress, making them particularly sensitive to salinity [25]. This findings of the present study demonstrated that salt stress significantly inhibited root growth in celery plants. The inhibitive effects were intensified as the NaCl concentration increased from 50 to 200 mM (Figure 1E–I), indicating that celery plants are sensitive to salt stress. In addition, this study found that different concentrations of NaCl inhibited the growth and biomass accumulation of celery plants (Figure 1A–D), which is consistent with previous studies reporting the effects of salt stress in rice [26], cucumber [27], tomato [28], and sweet pepper [5]. Based on the results obtained, the studies were conducted with 100 mM NaCl, which is considered moderate salt stress.

### 3.2. Exogenous Application of Trehalose Mitigated the Salinity-Induced Effects on Growth of Celery

Research has highlighted the potential positive effects of trehalose on regulating plant growth and development, including seed germination, flowering, seedling growth, and stomatal conductance [29]. Additionally, trehalose exhibits osmoprotectant characteristics that can induce salt tolerance in plants [11]. Results from the pesent study showed that trehalose application increased the plant height, maximum leaf length, stalk thickness, dry and fresh weight, and root morphology of celery plants under salt stress, but at varying degrees depending on the concentration. These parameters exhibited an initial increase followed by a decreasing trend with the increase in trehalose concentrations (Figure 2A–D, Table 1), suggesting that the optimal concentration 10 mM trehalose can substantially mitigate the inhibitory effects of salt stress on celery growth. F Kosar et al. reported that a small amount of exogenous application of trehalose could alleviate the adverse effects of abiotic stress on the physiological and biochemical characteristics of various plants, and the results of this study are consistent with them [10]. Similalry, foliar application of trehalose has been reported to alleviate the inhibitory effects of salt stress on growth of tomato [16] and rice [13]. Samadi et al. highlighted that application of 30 mM trehalose can significantly alleviate the inhibitory effect of salt stress on strawberry growth [30]. The results of principal component analysis (PCA) and membership function comprehensive evaluation analysis further confirm that trehalose has a positive alleviating effect on the growth of celery under salt stress (Figure 3B, Table 2).

### 3.3. Exogenous Application of Trehalose Enhanced the Photosynthesis Characteristics of Celery Under Salt Stress

Chloroplasts are the main sites for photosynthesis in plants and are also one of the most salt-sensitive organelles in plant cells [31]. Chlorophyll is the primary pigment that absorb light energy which is transform into organic matter during photosynthesis, simultaneously releasing energy. This process serves as the basic source of energy and nutrients for plants [32]. The findings of this study demonstrate that salt stress markedly decreased the chlorophyll contents and adversely affected the photosynthetic rate and stomatal conductance in celery leaves, while simultaneously increasing intercellular carbon dioxide concentration, therby inhibiting the overall photosynthetic process. These results align closely with the findings of previous studies that highlighted a significant decrease in photosynthetic pigments in kidney beans (*Phaseolus vulgaris* L.) [32] and in cucumber [33] as salinity stress levels increased. The observed effect could be attributed to the salinity-induced osmotic stress and ionic toxicity, which reduce the uptake of essential nutrients, particularly iron, nitrogen, magnesium, and potassium, crucial for chlorophyll biosynthesis. In addition, net photosynthetic rate (Pn) is the most sensitive physiological indicator of plant response to salt stress [34]. In this study, salt stress markedly decreased the photosynthetic rate compared to that of control. Chen et al. [35] reported that salt stress result in stomata closure due to osmotic stress. This stomata limitation, along with non-stomatal limiting factors reduse the photosynthetic rates in plants. Previous studies indicated that a simultaneous decrease in intercellular carbon dioxide concentration and stomatal conductance primarily results in lower photosynthetic rates due to stomatal factors. Conversely, when intercellular carbon dioxide concentration increases while stomatal conductance decreases, non-stomatal factors are the primary cause of the observed effects. On the other hand, the salinity induced detrimental effects on photosynthetic pigments, Pn, Gs, and Tr were significantly alleviated with the exogenous application of trehalose. In addition, it also reduced intercellular carbon dioxide concentration, which is consistent with the findings reported in tomatoes [16], rice [13], and strawberries [30]. It can be inferred that after exogenous application of trehalose, celery plants appear to regulate the transport of minerals by altering transpiration rates, thus reducing the adverse effects of salt stress. Furthermore, it can alleviate the inhibition of photosynthesis in celery leaves caused by salt stress through the regulation of non-stomatal factors. The light energy absorbed by chlorophylls is primarily utilize through three pathways, including the photosynthetic electron transport, chlorophyll fluorescence, and thermal dissipation.

Chlorophyll fluorescence parameters as a non-destructive probe for detecting photosynthesis, and any non-biotic stress affecting photosynthesis can be reflected through chlorophyll fluorescence parameters [16,36]. PSII is recognized as a primary site of damage to photosynthetic system caused by adverse environmental conditions and is crucial for light energy and electron transport in photosynthesis [36,37]. The Fv/Fm reflects the quantum yield when all photosystem PSII reaction centers are in the open state, serving as a direct indicator of primary photochemical efficiency [38]. The qP stands for photochemical quenching coefficient, reflecting the share of light energy captured by the pigment of the PSII antenna for photochemical electron transfer. Wu et al. [39] observed a decrease in Fv/Fm, Y(II), and qP in eggplant seedlings subjected to salt stress, accompanied by an increase in NPQ. Results from the present study also indicated comparable reduction in Fv/Fm, Y(II), qP, and Fv’/Fm’ in celery under salt stress. However, these detrimental effects were alleviated by exogenous trehalose application (Figure 5B–H), aligning with the results observed in tomatoes [16]. These findings indicate that salt stress adversely impacts the potential active centers of PSII in celery leaves [16], and the decline in Y(II) subsequently affects the synthesis of assimilation power, including ATP and NADPH, leading to a decrease in the rate of photosynthetic electron transport [39]. The decrease in qP suggests that electron flow from the donor side pf PSII to its reaction center is inhibited, resulting in a partial reduction in the energy supply for photochemical reactions and inhibition of the light reaction [24]. A previous study report has shown that exogenous application of trehalose enhances the efficiency of photochemical electron transport and the formation of photochemical energy, thereby increasing the number of electrons available for photochemical reactions and reducing the dissipation of light energy through heat or other mechanisms [16]. This effectively alleviates the salinity-induced damage to the photosynthetic apparatus of celery and maintains the normal function of PSII, which is in agreement with the findings of the previous studies [16,17,38]. The results of PCA and comprehensive evaluation of membership function analysis further confirmed the positive regulatory effect of trehalose on photosynthetic pigments, gas exchange parameters, and chloroplast fluorescence parameters under salt stress (Figure 6B, Table 3).

### 3.4. Regulatory Effect of Exogenous Trehalose on Photosynthetic System Performance of Celery Under Salt Stress

This study have shown that salt stress causes changes in the shape of the OJIP curve, as indicated by a slight decrease in overall fluorescence intensity (Figure 7A), which is consistent with the findings in tomatoes [40]. Two possible reasons have been suggested, firstly, salt stress reduces the reduction rate of the rapid reduction in the PQ pool during electron transfer, leading to a decrease in the I value [35]. Secondly, salt stress disrupts the chlorophyll proteins on the acceptor side of PSI or reduces the number of RC in PSII, consequently leading to a reduction in the *p* value [41]. The application of trehalose resulted in an increase in the overall fluorescence intensity (Figure 7A), which is consistent with the findings in tomatoes [16,35], indicating that trehalose alleviated the damage caused by salt stress to the OJIP curve of celery. The Vi and Vj values represent the relative variable fluorescence intensities at points I and J, respectively [42]. An increase in Vi indicates a decrease in the ability of the PQ pool to accept electrons, while a higher value of Vj reflects a lower efficiency of electron transfer from the primary quinone acceptor (QA) to the secondary quinone acceptor (QB) [43]. The value of dV/dto reflects the maximum rate of reduction in QA during photosynthetic electron transfer [16].

The photosynthetic performance index (PIabs) as an indicator of photosynthetic function and is highly sensitive to abiotic stresses [24]. This study showed that exogenous application of trehalose significantly decreased the Vj and dV/dto values, while increased PIabs (Figure 7B). This indicates that trehalose application increased the efficiency of electron transfer from QA to QB downstream in the photosynthetic electron transport chain, as well as the energy transfer from antenna absorption to QB and subsequent components [25], thereby accelerating electron transfer and enhancing photosynthetic capacity [43]. Furthermore, the results of this study indicate that optimal concentrations of trehalose enhance the absorption of light energy per unit area (ABS/CSm), the yield of electron transport per unit area (ETo/CSm), and the energy captured per reaction center for the reduction in QA (TRo/RC) under salt stress condition, while simultaneously decreasing heat dissipation per unit area (DIo/CSm) (Figure 8A,B). These observations suggests that trehalose protects the activity of the PSII reaction centers in celery under salt stress by increasing the energy absorbed, captured, and utilized for electron transport, while decreasing the energy dissipated [16]. We hypothesized that trehalose may enhance the ability of the photosynthetic process to withstand salt stress while also facilitating the conversion of the additional energy into electron transport, which are supported by the previous studies [37,43]. The comprehensive evaluation analysis and PCA of subordinate functions also verified the positive regulatory effects of trehalose on electron transfer in celery under salt stress (Figure 9B, Table 4).

### 3.5. Comprehensive Effects of Exogenous Trehalose on Various Indicators in Celery Under Salt Stress

To comprehensively and accurately assess the effects of exogenous trehalose on the growth and photosynthetic characteristics of celery under salt stress, the comprehensive analysis and ranking of all indicators were conducted using the membership function comprehensive evaluation analysis. The findings indicated that the treatment order was T5 > T4 > CK > T2 > T3 > T1 > N (Figure 10), with salt stress treatment at the end. Therefore, it can be inferred that exogenous trehalose positively regulated the growth and photosynthetic characteristics of celery under salt stress.

## 4. Materials and Methods

### 4.1. Plant Materials, Growth Conditions, and Treatments

The experiment was conducted in the greenhouse of Gansu Agricultural University, Lanzhou, northwestern China (36°05′ 39.86′ N, 103°42′ 31.09′ E). The celery variety (American celery) used in this experiment was acquired from Jiayuguan Baoneng Agricultural Technology Co., Ltd. (New District of Jiayuguan City, China). Trehalose was procured from Shanghai McLean Bio-Chemical Technology Co., Ltd. (Shanghai, China). Initially, the celery seeds were sown in plastic trays covered with a mixture of peat and perlite. After the emergence of two leaves and a terminal leaf, uniformly growing seedlings were selected and transplanted into plastic pots (volume, 6.28 L), filled with a growing medium consisting of cultivation substrate, grass charcoal and vermiculite (3:1:1 *v*/*v*/*v*).

After 7 days of transplanting, celery seedlings were foliar sprayed with different concentrations of trehalose (Tr) (Tr; 0, 1, 5, 10, and 15 mM) once a day at 09:00 am, and continued for 7 consecutive days. Subsequently, salt stress (100 mM) was imposed by providing 300 mL NaCl solution to each pot, repeated three times, each with an interval of three days. The salt stress level (100 mM) used in this study was based on our preliminary experiment using different NaCl concentrations (0, 50, 100, 150, and 200 mM). The 100 mM NaCl stress exhibited moderate negative effects on the growth of celery seedlings. Overall, the experiment consist of seven treatments: CK (0 mM Tr + 0 mM NaCl), N (0 mM Tr + 100 mM NaCl), T1 (1 mM Tr + 100 mM NaCl), T2 (5 mM Tr + 100 mM NaCl), T3 (10 mM Tr + 100 mM NaCl), T4 (15 mM Tr + 100 mM NaCl) and T5 (20 mM Tr + 100 mM NaCl). The treatments were organized in a completely randomized design, each including three replications and 15 seedlings in each replicate. After 30 days of transplanting, various growth and photosynthetic parameters were assessed.

### 4.2. Determination of Growth Indices

The height of celery plant and maximum leaf blade length were measured using a ruler. Stalk diameter was measured using a vernier caliper. Plant fresh weight was determined using a precision digital balance with an accuracy of one ten-thousandth of a gram. After washing the roots, images were captured using a root scanner (Perfection V700 Photo; EPSON, Beijing, China), and root morphological indices were analyzed using the WinRHIZO 5.0 software (WinRHIZO Pro LA2400, Regent Instruments Inc, Québec City, Canada). Then, the aboveground and underground parts of the plants were placed in a 105 °C oven for blanching for 30 min, followed by drying at 85 °C for one week. Finally, the dry weight was measured.

### 4.3. Determination of Photosynthetic Pigment and Gas-Exchange Parameters

The chlorophyll content was determined by the acetone extraction method [38]. Fresh leaves were collected from the celery plants and homogenized in 80% acetone. The absorbance of the reaction mixture was measured at 440, 645, and 663 nm using a spectrophotometer.

The gas exchange parameters including net photosynthetic rate (Pn), stomatal conductance (Gs), transpiration rate (Tr), and internal dioxide concentration in the intercellular spaces of the leaf blade (Ci) were measured using a portable photosynthesis meter (CIRAS2, PPsystem, Massachusetts, UK). The instrument parameters were set as follows: photosynthetically active radiation intensity of 1000 μmol m^−2^ s^−1^, carbon dioxide concentration of 380 μmol mol^−1^, and relative humidity of 75%.

### 4.4. Determination of Chlorophyll Fluorescence Parameters

The chlorophyll fluorescence parameters of leaves were measured using a modulated chlorophyll fluorescence imaging system (Walz, Effeltrich, Villingen-Schwenningen, Germany), as described by Tang et al. [37]. Three celery plants were randomly selected for each treatment group. Following 30 min of dark adaptation, functional leaves from the same plant part were excised, arranged, and fixed on the measurement platform of the fluorescence system. During measurement, the intensities of the detection light, photochemical light, and saturating pulse light were set at 0.1, 111, and 2700 μmol m^−2^ s^−1^, respectively. The duration of pulse light was 0.8 s, with a 20 s interval, resulting in a total of 15 measurements. Following the establishment of parameters, the initial fluorescence (Fo) and maximum fluorescence (Fm) under dark conditions were measured using the saturating pulse light, and the maximum photochemical efficiency of PSII (Fv/Fm) was calculated. After continuous exposure to photosynthetic light for 300 s, steady-state fluorescence was obtained. Meanwhile, the light-adapted maximum fluorescence yield (Fm′) was acquired after saturated pulsed light for 0.8 s. Eventually, when the actinic light (AL) was turned off and the far-red light was turned on, the light-adapted minimal fluorescence (Fo′) was obtained. The photochemical quenching coefficient (qP) and the actual photochemical efficiency of PSII (Y(II)) are calculated using the relevant formulas.

Fv/Fm=(Fm−Fo)/Fm

Y(II)=((Fm′−Fs)/Fm′)

qP=(Fm′−Fs)/(Fm′−Fo′)

qN=(Fm−Fm′)/(Fm−Fo′)

### 4.5. Determination of Kinetic Parameters for Rapid Chlorophyll Fluorescence Induction

The rapid chlorophyll fluorescence induction parameters were measured using a plant efficiency analyser (Handy PEA, Hansatech Instruments Ltd., King’s Lynn, UK) [37]. The calculation formula presented in Table 1. Before measurement, the leaves were dark-adapted for 30 min. Thereafter, the leaves were exposed to 3000 μmol/(m^2^ s) of red light for a duration of 2 s, and the rapid chlorophyll fluorescence induction kinetic curve (0-J-I-P fluorescence induction curve) was recorded. Additionally, various fluorescence parameters were calculated using the O-J-I-P test analysis method, based on the measured rapid chlorophyll fluorescence induction kinetic curve [38]. Table 5 Formulae and terminology used in the analysis of the OJIP fluorescence induction dynamics curve.

### 4.6. Calculation of Affiliation Function

The formulas used for the affiliation function method were:

Affiliation value=(X−X min)/(X max−X min)

Value of the inverse affiliation function=1−(X−X min)/(X max−X min)

where X is the measured value, X max is the maximum value, and X min is the minimum value.

### 4.7. Statistical Analysis

First, we evaluated the uniformity of data variance and the normality of dataset distribution. The distribution of all datasets was normal. Then, analysis of variance (ANOVA) was conducted using SPSS 20.0 software (SPSS Institute Inc., Chicago, IL, USA). Duncan’s multiple range test was used to compare the significant differences in the treatment methods at a probability level of 0.05. All data were collated using Excel 2020 (Microsoft Corporation Lake, Redmond, WA, USA). The experiment was conducted three times, and the results were expressed as mean ± standard error. All figures were constructed using Origin Pro 2022 software (Origin Lab Corporation, Northampton, MA, USA).

## 5. Conclusions

The findings from this study advocated that increase in salt stress level markedly inhibited the growth and photosynthetic attributes of celery. Under moderate salt stress (100 mM NaCl), pretreatment with different concentrations of trehalose mitigated the detrimental effects of salinity stress by reducing the excess excitation energy of photosystem II (PSII) in celery leaves through the heat dissipation pathway, alleviated the excitation pressure on the reaction center, increased the activity of the PSII reaction center per unit cross-sectional area, thereby improving the overall photosynthetic capicity, and improved the growth of celery under salt stress, and the most promising concentration of trehalose was 10 mM. Therefore, appropriate concentration of trehalose could be used as a potential strategy for expanding the celery cultivation on saline soil, and improving the productivity. Future studies will be conducted on the regulatory effect of trehalose on celery growth.

## Figures and Tables

**Table 1:** Effects of different concentrations of trehalose on root parameters of celery under salt stress.

Treatments	Total Root Length (cm)	Total Root Surface (cm^2^)	Total Root Volume (cm^3^)	Tips (Number)	Branching
CK	1972.10 ± 67.36 a	286.33 ± 6.23 b	2.78 ± 0.12 b	1663.67 ± 23.13 a	10785.33 ± 494.73 a
N	806.34 ± 10.35 d	128.27 ± 5.55 e	1.36 ± 0.05 c	592.33 ± 9.13 c	2734.33 ± 149.34 d
T1	1017.126 ± 14.25 bc	137.01 ± 5.15 de	1.47 ± 0.08 c	924.67 ± 21.37 b	4953.33 ± 142.54 bc
T2	1163.70 ± 63.57 b	153.38 ± 5.28 cd	1.61 ± 0.03 c	904.67 ± 23.68 b	5779.67 ± 398.52 b
T3	2113.12 ± 43.36 a	318.12 ± 10.56 a	3.83 ± 0.52 a	1701.00 ± 35.53 a	11592.33 ± 293.58 a
T4	1153.55 ± 50.50 b	163.01 ± 4.15 c	1.83 ± 0.06 c	974.00 ± 24.58 b	5274.33 ± 292.59 bc
T5	954.07 ± 64.38 cd	133.91 ± 6.38 de	1.50 ± 0.07 c	946.00 ± 20.22 b	4388.67 ± 125.42 c

Data are the means ± SE (*n* = 3). Different letters indicate significant differences among treatments based on the Duncan’s test (*p* < 0.05). “Tips” represent the number of root tips, and “branching” represents the number of root branching.

**Table 2:** Comprehensive evaluation analysis and ranking of morphological indicator affiliation functions.

Value of the Affiliation Function							
	CK	N	T1	T2	T3	T4	T5
X1	0.4924	0.3733	0.6429	0.4242	0.6264	0.6190	0.4686
X2	0.5889	0.4047	0.4012	0.4520	0.5778	0.3556	0.3636
X3	0.4333	0.5034	0.3905	0.6385	0.6044	0.5417	0.5924
X4	0.5778	0.4545	0.3704	0.5889	0.4127	0.4218	0.5208
X5	0.3845	0.5281	0.5292	0.5493	0.4491	0.5805	0.5398
X6	0.4762	0.5000	0.4091	0.6061	0.3810	0.5000	0.4444
X7	0.4264	0.5263	0.3385	0.5000	0.5526	0.3868	0.5631
X8	0.5029	0.4162	0.5054	0.4368	0.3912	0.5588	0.4796
X9	0.4331	0.4222	0.3671	0.4618	0.4399	0.4500	0.3497
X10	0.6151	0.6147	0.5958	0.5333	0.4616	0.5122	0.5823
X11	0.4515	0.5591	0.5090	0.4060	0.5537	0.6364	0.5507
X12	0.5382	0.5897	0.3801	0.5585	0.5315	0.3637	0.4691
Average	0.4933	0.4913	0.4533	0.5129	0.4985	0.4942	0.4937
Ranking	5	6	7	1	2	3	4

**Table 3:** Comprehensive evaluation analysis and ranking of photosynthetic parameters and chlorophyll fluorescence parameters.

Value of the Affiliation Function							
	CK	N	T1	T2	T3	T4	T5
Y1	0.4741	0.6222	0.3785	0.6401	0.3856	0.4646	0.4422
Y2	0.6565	0.4157	0.5265	0.5152	0.4888	0.4642	0.4313
Y3	0.5043	0.4360	0.5947	0.4511	0.4127	0.6616	0.4392
Y4	0.4444	0.4103	0.3810	0.5000	0.4500	0.5926	0.5758
Y5	0.5833	0.4444	0.5556	0.4000	0.3889	0.4000	0.4074
Y6	0.6222	0.4000	0.5714	0.4052	0.5128	0.5926	0.6190
Y7	0.5667	0.5185	0.3333	0.5778	0.4568	0.5897	0.6190
Y8	0.4074	0.6667	0.3333	0.6667	0.3810	0.5833	0.6000
Y9	0.6111	0.3768	0.6611	0.3333	0.4615	0.5000	0.5139
Y10	0.3675	0.6286	0.6460	0.3889	0.4167	0.6357	0.6000
Y11	0.6087	0.6047	0.6395	0.3333	0.5965	0.3704	0.3333
Y12	0.6087	0.6047	0.6395	0.3333	0.5965	0.3704	0.3333
Y13	0.5131	0.6321	0.4348	0.3862	0.5758	0.6235	0.6159
Y14	0.6394	0.4019	0.5001	0.5164	0.3473	0.6605	0.5391
Average	0.5434	0.5116	0.5140	0.4605	0.4622	0.5364	0.5050
Ranking	1	4	3	7	6	2	5

**Table 4:** Comprehensive evaluation and ranking of membership functions related to linear electron transport characteristics in PSII.

Value of the Affiliation Function							
	CK	N	T1	T2	T3	T4	T5
Z1	0.3578	0.4167	0.5067	0.4672	0.5931	0.5321	0.5350
Z2	0.5784	0.3561	0.3346	0.5611	0.5418	0.6402	0.5967
Z3	0.3445	0.4222	0.6179	0.5712	0.5679	0.4507	0.3924
Z4	0.5464	0.5404	0.5784	0.5615	0.5580	0.6049	0.6152
Z5	0.6231	0.3335	0.4561	0.4808	0.4462	0.5203	0.3920
Z6	0.5041	0.5079	0.6246	0.5889	0.6481	0.3521	0.5775
Z7	0.4074	0.5333	0.3922	0.5926	0.5370	0.6667	0.5287
Z8	0.5375	0.5208	0.6263	0.7230	0.5128	0.3529	0.5066
Z9	0.5833	0.4659	0.3750	0.6190	0.4928	0.3499	1.000
Z10	0.3494	0.4590	0.5354	0.3358	0.4786	0.5887	0.6043
Z11	0.6552	0.4171	0.4913	0.4366	0.4596	0.4063	0.3655
Z12	0.3579	0.4674	0.5541	0.5470	0.3595	0.3814	0.6661
Z13	0.3526	0.5127	0.4286	0.4871	0.5778	0.5180	0.5635
Average	0.4767	0.4579	0.5016	0.5363	0.5210	0.4896	0.5649
Ranking	6	7	4	2	3	5	1

**Table 5:** Formulae and terms used in the analysis of the OJIP fluorescence induction dynamics curve [25].

Formulae and Terms	Description
Fo	Minimal recorded fluorescence intensity
Fm	Maximal recorded fluorescence intensity
Vj = (FJ − Fo)/(Fm − Fo)	Relative variable fluorescence intensity at the J-step
Vi = (F30 ms − Fo)/(Fm − Fo)	Relative variable fluorescence intensity at the I-step
φEo = ETo/ABS = [1 − (Fm/Fm)]·ψo	Quantum yield for electron transport (at t = 0)
PIabs = (RC/ABS)·[φPo/(1 − φPo)]·[ψo/(1 − ψo)]	Performance index on absorption basis
ABS/CSm ≈ Fm	Absorption flux per cross section
TRo/CSm ≈ φPo·(ABS/CSm) = Fm·[1 − (Fo/Fm)]	Trapped energy flux per PSII cross section
ETo/CSm ≈ Fm·[1 − (Fo/Fm)]·(1 − Vj)	Electron transport in PSII cross section
DIo/CSm ≈ (ABS/CSm) − (TRo/CSm)	Dissipated energy flux per PSII cross section
ABS/RC = Mo·(1/Vj)·(1/φPo)	Absorption flux per RC
TRo/RC = Mo·(1/Vj)	Trapped energy flux per RC
ETo/RC = Mo·(1/Vj)·ψo	Electron transport flux per RC
DIo/RC = (ABS/RC) − (TRo/RC)	Dissipated energy flux per RC

## Data Availability

All data, tables and figures in this manuscript are original.
